# Association between dietary inflammation and erectile dysfunction among US adults: A cross-sectional analysis of the National Health and Nutrition Examination Survey 2001–2004

**DOI:** 10.3389/fnut.2022.930272

**Published:** 2022-11-11

**Authors:** Zhijie Ruan, Xiaoping Xie, Haoyang Yu, Ruimin Liu, Wenjuan Jing, Tao Lu

**Affiliations:** Department of Dermatology, First Affiliated Hospital of Shantou University Medical College, Shantou, China

**Keywords:** erectile dysfunction, inflammation, dietary score, dietary recall, NHANES, cross-sectional study

## Abstract

**Background:**

Although chronic low-grade inflammation has been linked to the development of erectile dysfunction (ED), the association between pro-inflammatory diets and ED is unclear. The dietary inflammation index (DII) is a novel method to quantify the inflammatory potential of a diet.

**Objective:**

Our objective was to investigate the association between the DII and ED among US males.

**Design:**

This cross-sectional study included 3,693 males 20–85 year of age from the National Health and Nutrition Examination Survey (NHANES) 2001–2004. Multivariable-adjusted logistic regression models were used to assess the association between the DII and ED. All analyses accounted for the complex sampling design.

**Results:**

The mean ± SE of the DII was 0.8 ± 0.1 and 0.4 ± 0.1 among participants with and without ED, respectively. After adjusting for age, race/ethnicity, education, smoking status, physical activity, drinking status, hypertension, diabetes, cardiovascular disease, hypercholesterolemia, BMI, and eGFR, the DII score was associated with ED (odds ratio 1.12; 95% CI: 1.04–1.19). Moreover, this association was also stable in our subgroup analysis or sensitivity analyses.

**Conclusion:**

Dietary inflammatory potential, as estimated by the DII score, is positively associated with ED among US males.

## Introduction

Erectile dysfunction (ED) is one of the most common types of sexual dysfunction in men (1, 2) and is predicted to affect approximately 322 million men worldwide by 2025 (3). ED has led to a poorer quality of life and reduction of economic productivity in males, as well as a substantial financial burden on society (4, 5). Some ED risk factors have been identified, including aging, history of diabetes, cardiovascular disease, chronic kidney disease, and smoking (6, 7). However, those risk factors, in most cases, are limited or unmodifiable. Thus, the identification of modifiable risk factors for ED is important.

Diet is a potential source of chronic low-grade inflammation which is related to the pathogenesis of ED (8). Previously, some dietary patterns, especially anti-inflammatory diet, have been linked to ED (9, 10). For example, Mediterranean diet is a kind of anti-inflammatory diet, and recently a long-term randomized clinical trial by Maiorino et al. has noticed a protective effect of Mediterranean diet on erectile function, as well as a reduction of C-reactive protein levels in males with newly diagnosed type 2 diabetes (T2DM) (11). It can be inferred that dietary inflammatory potential is related to the development of ED. However, the impact of the dietary inflammatory potential on ED is still unclear. The DII is a novel method re-devised by Shivappa et al. to quantify the potential inflammatory levels of our daily diet (12). Since its development, DII has been validated against many inflammatory biomarkers in different ethnics and was widely used in a large number of studies (13–19). To our knowledge, the association between the level of dietary inflammation potential and ED has not been reported before. To best understand the influence of dietary inflammation on ED and provide clues for its prevention, this cross-sectional study explored the association between DII and ED among US adults, using data from the National Health and Nutrition Examination Survey (NHANES).

## Materials and methods

### Data sources

The NHANES is a nationally representative survey with a stratified, multistage probability cluster sampling design in the USA. It is administered by the National Center for Health Statistics (NCHS) and can be used to assess the health or nutritional status of the non-institutionalized US population (20). Data for erectile function is available in the NHANES 2001–2004 and all data were collected following standardized protocols from the NCHS. To provide reliable estimates, we utilized the 4-year data for analyses. The study was approved by the NCHS Research Ethics Review Board and written consents were obtained from the participants before their participating. We followed the Strengthening the Reporting of Observational Studies in Epidemiology – Nutritional Epidemiology (STROBE-nut) guidelines in reporting.

### Study population

**FIGURE 1:**
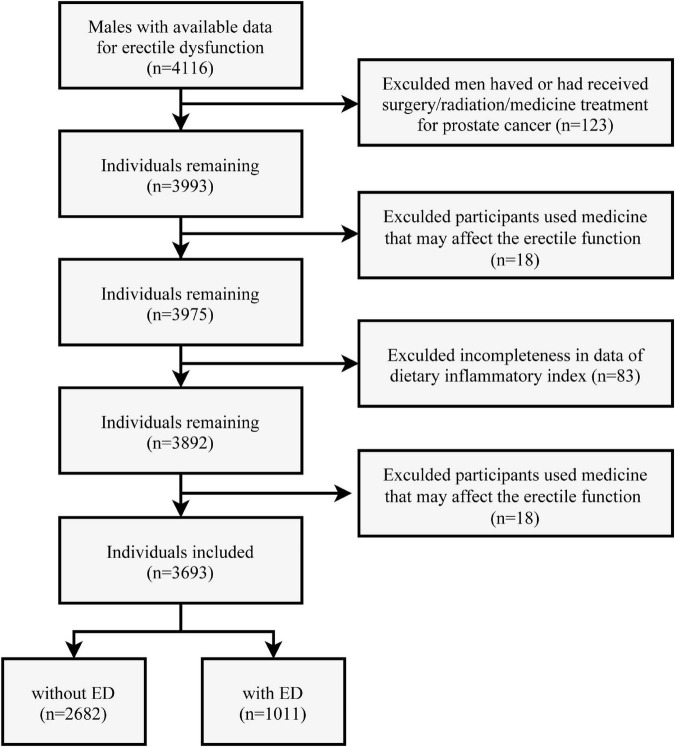
Flow diagram of the screening and enrollment of study participants.

Of 4116 males (≥20 years) with available information for self-reported ED in the NHANES 2001–2004, 125 were excluded due to having or having received surgery/radiation/medicine treatment for prostate cancer. Eighteen men were further excluded for taking phosphodiesterase type 5 (PDE5) inhibitors or Yohimbine (21), and 83 participants were excluded due to incompleteness of data needed to calculate the dietary inflammatory index. According to a previous study, a daily calorie intake below 800 kcal or above 5,000 kcal is thought to be implausible (22). Thus, 199 participants were excluded for this reason. The flow chart for subject selection is presented in Figure 1.

### Definition of erectile dysfunction and dietary inflammation index

In the NHANES, erection function was assessed by a question: “How would you describe your ability to get and keep an erection adequate for satisfactory intercourse?” The following response options were provided: “always or almost always able,” “usually able,” “sometimes able,” or “never able.” This single question has been validated in a sub-sample from the Massachusetts Male Aging Study (MMAS) and was considered as a practical tool for assessing ED (23). In the present study, ED was defined as a dichotomous variable where men who responded “sometimes able” or “never able” to maintain an erection were considered as with ED and those who responded “always or almost always able” or “usually able” were considered as without ED (24, 25).

Based on the previous study, the DII score was calculated based on 27 food parameters extracted from the NHANES 2001–2004 by a 24-h dietary recall interview (12, 26). Then, the DII score was calculated by the following steps. First, the *z*-score for each of the food parameters for each individual was calculated based on the world average and standard deviation. Second, to control the effect of “skewing,” each *z*-score was converted to a centered percentile value. Third, the food parameter-specific DII score was calculated as the centered percentile value times its respective standardized overall inflammatory effect score (12). Finally, the parameter-specific DII scores were summed to get the final index for each individual. A more positive DII value indicates a more pro-inflammatory diet, and a more negative DII value indicates a more anti-inflammatory diet. Supplementary Table 1 lists the respective world average, SD, and standardized overall inflammatory effect score of the 27 food parameters used in the present study.

### Covariates

Potential confounders in the present study included age (27), race/ethnicity (non-Hispanic white, Mexican-American, non-Hispanic black, and others) (28), education (high school or less, some college, and college graduate or higher) (29), body mass index (BMI) (30), diabetes (31), hypertension (32), cardiovascular disease, hypercholesterolemia (33), estimated glomerular filtration rate (eGFR) (34, 35), smoking status (36), alcohol drinking status (37), and physical activity level (38). Diabetes was defined as any participant with self-reported diabetes or who had a fasting plasma glucose level of 126 mg/dl or greater or a glycated hemoglobin level of 6.5% or greater. Hypertension was defined as taking anti-hypertensive agents, systolic blood pressure ≥140 mmHg, or diastolic blood pressure ≥90 mmHg. Cardiovascular disease was defined as self-reported history of one of the following conditions: coronary heart disease, myocardial infarction, congestive heart failure, and stroke. Hypercholesterolemia was defined as being told to take cholesterol-lowering medications or total cholesterol ≥240 mg/dl. The eGFR was calculated based on the Chronic Kidney Disease Epidemiology Collaboration (CKD-EPI) equation. In the NHANES, self-reported smoking status can be assessed by the following two survey question: “Have you smoked at least 100 cigarettes in your entire life?” and “Do you now smoke cigarettes every day, some days or not at all?” According to the National Health Interview Survey, smoking status can be categorized into three groups: never smoker (smoked less than 100 cigarettes in the lifetime, or has never smoked), ex-smoker (smoked at least 100 cigarettes in the lifetime and responded that now do not smoke), current smoker (smoked at least 100 cigarettes in the lifetime and responded that now smoke cigarettes every day or some days) (39). Serum cotinine is a biomarker of current smoking. Considering that there is no minimum duration of smoking cessation, we took serum cotinine into consideration to reduce the misclassification of current smoker into ex-smoker. Specifically, self-reported never smokers and ex-smokers who having a serum cotinine level above 10 ng/ml were corrected to be current smokers (40). Alcohol drinking status was determined by self-reporting. Those who drank at least 12 standard drinks in any one year were defined as “alcohol drinking,” otherwise they were designated as “without alcohol drinking.” Physical activity was divided into three groups based on self-reported leisure-time physical activity: inactive, moderate, and vigorous.

### Statistical analysis

The sampling weights were applied in our analyses following the NHANES analytic guidelines (41). A 4-year sampling weight was calculated using the formula: Dietary day one 4-Year sample weight = 1/2 × Dietary day one 2-Year sample weight (WTDRD1). Weighted means/proportions and standard errors (SEs) were used to describe the characteristics of the participants. Continuous data were compared using the survey *t*-test, and categorical data were compared by the survey (Rao–Scott) χ^2^ test. Since the missing data was small (missing rate ranged from 0 to 3.9%) for any variable, no imputation method was used in the present study. Odds ratio (OR) and 95% confidence interval (CI) were calculated to show the association between DII and ED by using logistic regression models. Four models were conducted using the logistic regression analyses and generalized variance-inflation factors (GVIF) ≥3 indicated the presence of multicollinearity in the analysis. Model 1 was the crude model with no covariate adjusted. Model 2 was adjusted for age, race/ethnicity. Model 3 was the main model. If a covariate changed the estimates of DII and ED by more than 10% when entered into the crude model or eliminated from the complete model, it was included as a potential confounder in model 3. Therefore, model 3 was adjusted for age, race/ethnicity, education levels, smoking status, physical activity levels and hypertension. A fully adjusted model was done for model 4, which was adjusted for covariates in model 3 and drinking status, diabetes, cardiovascular disease, hypercholesterolemia, BMI, and eGFR. To further explore the potential associations, the DII score was also classified by tertiles for multivariable logistic regression analyses, and tests for trend were conducted by entering the median value of each DII tertiles as a continuous variable in the multivariable logistic regression models. Stratified and interaction analyses were performed according to age groups, race and ethnicity, hypertension, diabetes, and cardiovascular disease. Finally, two sensitivity analyses were additionally performed to assess the robustness of our findings. In the first sensitivity analysis, we excluded participants taking medicines that potentially affect erectile function, including antidepressants (42), antipsychotics (43), antihyperglycemic agents (9, 44), sex hormones and corticosteroids (45). In the second sensitivity analysis, a stricter criterion of ED was used. Only those who responded “never able to get and keep an erection adequate for satisfactory intercourse” were considered as having ED. All statistical analyses were performed with the statistical software R (The R Foundation)^1^. A *P*-value <0.05 (two-sided) was considered to indicate statistical significance. A *post hoc* power analysis was performed, which demonstrated that the power for the primary outcomes was sufficient (power >0.90).

## Results

Table 1 shows the weighted characteristics stratified by ED status. There were 3,693 males included in our analyses and 1,011 of them had ED. The weighted number of all participants is 3,809,255,599, and the weighted prevalence of ED is 33.7%. For all participants, the weighted mean age was 44.8 years old (SE = 0.4), and most of them were non-Hispanic whites (74.6%, SE = 2.0). The DII scores ranged from −5.15 (most anti-inflammatory) to +4.93 (most pro-inflammatory), and the mean of DII score was higher in participants with vs. without ED (0.8 vs. 0.4, *P* < 0.001). Participants with ED were more likely to be older and have a higher BMI, lower educational level, lower eGFR, lower physical activity level, and diabetes, hypertension, cardiovascular disease, and hypercholesterolemia.

**TABLE 1:** Weighted distributions of characteristics of participants.^a^

	All participants		No ED		ED		*P*-value
	No.^b^	Mean ± SE	No.^b^	Mean ± SE	No.^b^	Mean ± SE	
AGE, year	3,693	44.8 ± 0.4	2,682	41.2 ± 0.3	1,011	61.4 ± 0.5	<0.001
BMI, kg/m^2^	3,607	28.1 ± 0.1	2,646	27.9 ± 0.2	961	28.9 ± 0.3	<0.001
DII	3,693	0.4 ± 0.1	2,682	0.4 ± 0.1	1,011	0.8 ± 0.1	<0.001
eGFR, [ml/(min × 1.73m^2^)]	3,550	93.2 ± 0.6	2,578	96.3 ± 0.6	972	79.8 ± 1.0	<0.001
Race/ethnicity, %							0.09
Mexican-American	774	7.7 ± 1.2	560	8.1 ± 1.2	214	6.1 ± 1.5	
Non-Hispanic black	681	9.9 ± 1.1	529	10.2 ± 1.2	152	8.4 ± 1.3	
Non-Hispanic white	1,994	74.6 ± 2.0	1,406	73.7 ± 2.0	588	78.4 ± 2.6	
Others	244	7.8 ± 1.1	187	7.9 ± 1.1	57	7.1 ± 1.5	
Education, %							<0.001
High school or less	1,023	16.4 ± 0.8	612	13.5 ± 0.8	411	29.1 ± 2.4	
Some college	915	25.9 ± 1.1	709	26.5 ± 1.3	206	23.4 ± 1.7	
College graduate or higher	1,753	57.7 ± 1.4	1,359	59.9 ± 1.4	394	47.5 ± 2.1	
Diabetes, %	513	10.2 ± 0.6	216	6.3 ± 0.6	297	27.6 ± 1.6	<0.001
Hypertension, %	1,247	30.8 ± 1.3	664	24.7 ± 1.3	583	57.5 ± 1.6	<0.001
Cardiovascular disease, %	489	9.7 ± 0.8	178	5.4 ± 0.7	311	29.0 ± 2.1	<0.001
Hypercholesterolemia, %	1,023	28.5 ± 1.1	646	25.3 ± 1.2	377	42.5 ± 2.5	<0.001
Alcohol drinker, %	3,068	82.6 ± 2.1	2,244	83.1 ± 2.3	824	80.4 ± 2.2	0.21
Smoker, %							<0.001
Never smoker	1,363	38.7 ± 1.8	1,087	41.5 ± 2.0	276	26.5 ± 2.0	
Ex-smoker	1,046	25.2 ± 1.1	594	21.4 ± 1.2	452	42.2 ± 2.1	
Current smoker	1,281	36.2 ± 1.8	1,000	37.1 ± 1.9	281	31.3 ± 2.9	
Physical activity, %							<0.001
Inactive	1,433	31.3 ± 1.2	915	28.1 ± 1.3	518	44.5 ± 2.1	
Moderate	1,004	28.8 ± 0.9	675	26.9 ± 1.1	329	37.1 ± 1.8	
Vigorous	1,256	39.9 ± 1.4	1,092	44.9 ± 1.6	164	17.8 ± 2.2	

BMI, body mass index; DII, dietary inflammation index; eGFR, estimated glomerular filtration rate; ED, erectile dysfunction.

^a^Data are presented as weighted percentages ± SE for categorical variables and weighted means ± SE for continuous variables.

^b^Unweighted numbers.

Table 2 summarizes results from sample-weighted logistic regression analyses. The association between DII and ED was stable in different adjusted models. In the crude model (model 1), the odds ratio of DII on ED was 1.13 (95% CI, 1.08–1.19). Males in the highest DII tertiles vs. those the lowest DII tertiles were at a higher risk of ED [OR 1.64 (95% CI, 1.30–2.08)]. In the main model (model 3) adjusted for age, race and ethnicity, education levels, hypertension, smoking status, and physical activity levels, the odds ratio was 1.11 (95% CI, 1.05–1.18). The odds ratios were 1.19 (95% CI, 0.85–1.66) and 1.47 (95% CI, 1.12–1.94) for DII tertiles 2 and 3, respectively (*p* for trend = 0.01). Furthermore, this association was stable in the fully adjusted model and the trend was robust.

**TABLE 2:** Association between DII and ED.

	Model 1^a^	Model 2^b^	Model 3^c^	Model 4^d^
DII (continuous)	1.13 (1.08, 1.19)	1.12 (1.05, 1.18)	1.11 (1.05, 1.18)	1.12 (1.04, 1.19)
DII categories				
Tertile 1	Ref.	Ref.	Ref.	Ref.
Tertile 2	1.34 (1.00, 1.78)	1.22 (0.88, 1.69)	1.19 (0.85, 1.66)	1.16 (0.80, 1.67)
Tertile 3	1.64 (1.30, 2.08)	1.48 (1.13, 1.93)	1.47 (1.12, 1.94)	1.51 (1.09, 2.10)
*P* for trend	<0.001	0.01	0.01	0.02

DII, dietary inflammation index; ED, erectile dysfunction.

^a^Model 1: crude model.

^b^Model 2: adjusted for age, race and ethnicity, and education.

^c^Model 3 (the principal model): model 2 + physical activity, smoking status, and hypertension.

^d^Model 4 (the fully adjusted model): model 3 + drinking status, diabetes, cardiovascular disease, hypercholesterolemia, BMI, and eGFR.

**FIGURE 2:**
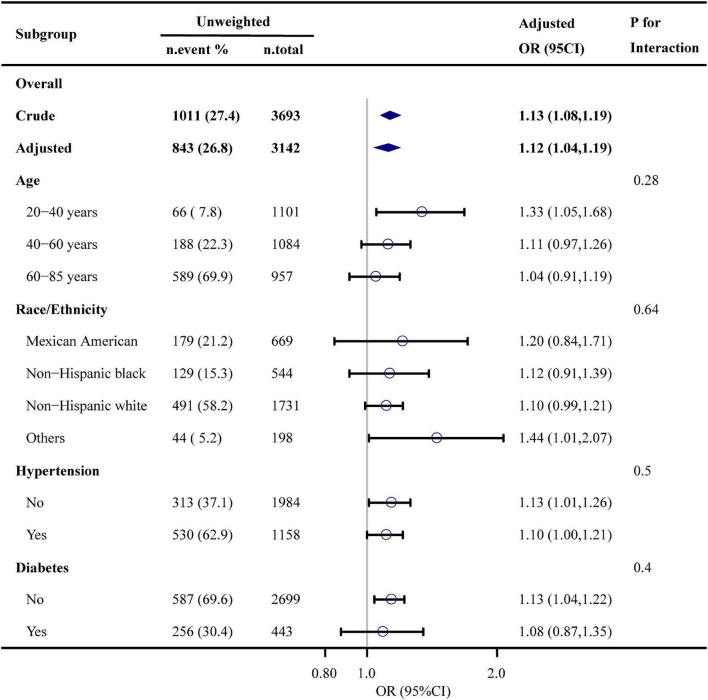
Association between dietary inflammation index and erectile dysfunction. Each stratification was adjusted for age, race and ethnicity, educational level, physical activity, smoking status, drinking status, BMI, hypertension, diabetes, cardiovascular disease, hypercholesterolemia, and eGFR, except the stratification factor itself.

Figure 2 shows the results of subgroup analysis. The DII score was associated ED among those aged 20 to 40 years (OR, 1.33; 95% CI, 1.05–1.68), and those with (OR, 1.10; 95% CI, 1.00–1.21) and without hypertension (OR, 1.13; 95% CI, 1.01–1.26), without diabetes (OR, 1.13; 95% CI, 1.04–1.22). No significant interaction was detected in the interaction analysis. Results of sensitivity analyses are presented in Table 3. After excluding participants taking medicines that potentially affect erectile function, the odds ratio was 1.11 (95% CI, 1.03–1.20) after adjusting for age, race and ethnicity, education levels, hypertension, smoking status, and physical activity levels. After re-defining ED to self-reported “never able” to maintain an erection, the odds ratio was 1.16 (95% CI, 1.06–1.27) in the adjusted model.

**TABLE 3:** Sensitivity analyses.

	DII	T1	T2	T3	*P* for trend
**Sensitivity analysis 1^a^**					
Crude model	1.14 (1.08, 1.21)	Ref.	1.33 (0.98, 1.81)	1.70 (1.30, 2.24)	<0.001
Adjusted model^b^	1.11 (1.03, 1.20)	Ref.	1.31 (0.88, 1.94)	1.53 (1.08, 2.17)	0.02
**Sensitivity analysis 2^c^**					
Crude model	1.18 (1.10, 1.25)	Ref.	1.86 (1.21, 2.86)	2.25 (1.67, 3.05)	<0.001
Adjusted model^b^	1.16 (1.06, 1.27)	Ref.	2.01 (1.24, 3.24)	2.12 (1.41, 3.18)	0.002

^a^Sensitivity analysis excluding participants taking medication that potentially affect erectile function.

^b^Adjusted for age, race and ethnicity, education, physical activity, smoking status, and hypertension.

^c^Sensitivity analysis that re-defines ED to self-reported “never able” to maintain an erection.

## Discussion

This nationally representative study found robust association between DII and ED in US adult males. In the present study, after adjusting for the baseline imbalance, participants in the highest DII tertiles still had an approximately 1.5-time higher odds of having ED compared with those without ED. Besides, this relationship remained stable in the full-adjusted model that additionally adjusted for a large set of covariates. Notably, the slight variations of the odds ratio between the full-adjusted model and model 2 may indicate that some risk factors of ED (like diabetes, cardiovascular disease, and hypercholesterolemia) may not play a key role here. However, it should be interpreted cautiously, due to the potential residual confounding. Interestingly, in our subgroup analyses, males who are younger and without diabetes seemed to have a higher risk of ED, although no significant interaction was detected. Considering the cross-sectional nature, this may be explained by the reverse causation. ED is a common situation in the elderly persons who are more prone to chronic diseases, like diabetes. The higher risk of developing many chronic diseases may encourage the old to choose a healthier dietary pattern which may content more anti-inflammatory component. In our sensitivity analysis that ruled out the impact of some medication on ED, the odds ratio remained. In the other sensitivity analysis that redefined ED by using a stricter criterion, we noticed a higher odds ratio. This could be explained by a reduction of the misclassification of cases into non-cases which can bias the odds ratio to null. To conclude, the present study provides evidence of a robust association between DII and ED.

Most existing studies focus on the protective effect of specific anti-inflammatory nutrients or diet patterns on ED. As listed in Supplementary Table 1, both caffeine and flavonoids are anti-inflammatory food parameters. A study by Lopez et al. noticed a protective effect of caffeine against the risk of developing ED (22), and Cassidy et al. reported a protective effect of flavonoids against the risk of developing ED (46). Current evidence also suggested an anti-inflammatory effect of vitamin D (47, 48), and Farag et al. found a positive association between vitamin D deficiency and ED (21). These studies support our findings very well. For pro-inflammatory dietary components, it is hard to judge its impact on ED since only limited relevant studies. Saturated fatty acids (SFA) and fat are widely considered as pro-inflammatory dietary components. Medeiros Júnior et al. has found that a high-SFA diet could led to an increase in collagen fibers and decline in corpus cavernosum cell proliferation in rat penile tissue (49). Nguyen et al. also found that a high-fat diet in combination with marijuana can lead to an accelerated corporal fibrosis in mouse (50). However, those are all indirect evidence and further studies are needed in this field. Both Mediterranean diet and plant-based diet are previously found to be inversely associated with DII score and they are also suggested to play a role in maintaining erectile health (51, 52). In 2010, Giugliano et al. found that a greater adherence to Mediterranean diet was associated with a lower risk of ED in Italian male that with T2DM (53). To further assess the effect of Mediterranean diet on ED, they conducted a long-term dietary trial in participants with newly diagnosed T2DM (11). It shows that over the entire follow-up, men in the Mediterranean diet group had a better ED as well as a lower C-reactive protein levels compared with those in the low-fat group. However, since those studies were conducted in participants with T2DM, application of the results to the general population should be carefully considered. Recently, a cross-sectional study by Carto et al. has reported that a healthful plant-based diet was negatively associated with ED among the US population (54). A cohort study by Yang et al. also found that healthy plant-based diet indices was inversely associated with incident ED and unhealthy plant-based diet indices was positively associated with incident ED among US elderly males (55). A possible explanation for those studies could be both of those two diet patterns share some similar anti-inflammatory food groups, which may have protective effect on ED. Results of our study complement the previous research and may provide valuable information for understanding the pathology of diet on ED.

Although previous studies have noticed an elevation of inflammatory biomarkers in both animal models and humans with ED (56, 57), the exact mechanism is still unclear. A possible explanation is that the pro-inflammatory diet mediated ED by increasing the vascular endothelial injury. As is known, endothelial nitric oxide is a molecule that regulates vascular tone and can protect endothelial cells from oxidative damage. Previous studies showed that inflammatory biomarkers, such as TNF-α, can inhibit endothelial nitric-oxide synthase (eNOS) gene expression in endothelial cells (58–60), leading to vascular endothelial injury and a higher risk of ED. In addition, a pro-inflammatory diet can contribute to the pathogenesis of diabetes and cardiovascular disease. Both are potential risk factors of ED.

The present study has serval strengths. First, it used large high-quality data from the NHANES and considered many potential covariates, which strengthens the reliability of the results. Furthermore, since all analyses were accounted for the NHANES complex sampling design, these findings are generalizable to general US males. Finally, to our knowledge, this is the first study to explore the association between DII and ED. It provides data for future studies. However, some limitations should be mentioned in this study. First, due to the cross-sectional nature, causal inference about the association between DII and ED could not be established. Thus, further well-designed cohort studies are needed for future studies. Second, although the single question to access ED was validated in the previous study, there could still be a recall bias. Thus, we also conducted a sensitivity analysis that re-defined ED by using a stringent criterion to further verify the reliability of our results. Third, although we adjusted for potential confounders as far as possible, there could be some residual or unmeasured confounders, such as glucocorticoid use (45). Thus, we conducted a sensitivity analysis that excluded participants taking medicines that potentially affect erectile function. Finally, the DII was calculated based on a 24-h dietary recall interview in the present study. Although a prospective investigation found that DII is relatively constant during several years of observation in females (61), it is not necessarily generalizable to males. Nevertheless, since the present study is the first one to investigate the DII–ED relationship, it still provides preliminary evidence in this direction.

## Conclusion

In summary, this cross-sectional analysis suggests that dietary inflammatory potential, as estimated by the DII score, is positively associated with ED in non-institutionalized US males. Since a pro-inflammatory diet may be a modifiable risk factor of ED, we expect more studies on this field.

## Data availability statement

Publicly available datasets were analyzed in this study. This data can be found here: CDC National Center for Health Statistics NHANES database: https://wwwn.cdc.gov/nchs/nhanes/Default.aspx.

## Author contributions

ZR contributed to study planning, data analyses, and drafting of the manuscript. XX, HY, RL, WJ, and TL contributed to study planning and manuscript development. All authors read and approved the final manuscript.

## Footnotes

1
http://www.R-project.org
