# Supplementary material for: Association between dietary inflammation and erectile dysfunction among US adults: A cross-sectional analysis of the National Health and Nutrition Examination Survey 2001–2004
Source: Front Nutr. 2022 Nov 11;9:930272. doi: 10.3389/fnut.2022.930272 (PMC9691656; doi:10.3389/fnut.2022.930272)
Supplement: Supplementary file 1 [file Table_1.DOCX]

Zhijie Ruan: Association between dietary inflammation index and erectile dysfunction among US adults: a cross-sectional analysis of the NHANES 2001-2004

**Supplementary Table1** Food parameters included in the dietary inflammatory index, inflammatory effect scores, and intake values from the global composite data set.

| Food parameter | Overall inflammatory effect score | Global daily mean intake (units/d) | SD |
| --- | --- | --- | --- |
| Carbohydrate (g) | 0.097 | 272.2 | 40 |
| Fibre (g) | -0.663 | 18.8 | 4.9 |
| Protein (g) | 0.021 | 79.4 | 13.9 |
| Total fat (g) | 0.298 | 71.4 | 19.4 |
| Cholesterol (mg) | 0.11 | 279.4 | 51.2 |
| n-3 Fatty acids (g) | -0.436 | 1.06 | 1.06 |
| n-6 Fatty acids (g) | -0.159 | 10.8 | 7.5 |
| MUFA (g) | -0.009 | 27 | 6.1 |
| PUFA (g) | -0.337 | 13.88 | 3.76 |
| Saturated fat (g) | 0.373 | 28.6 | 8 |
| Vitamin A (RE) | -0.401 | 983.9 | 518.6 |
| β-Carotene (μg) | -0.584 | 3718 | 1720 |
| Thiamin (mg) | -0.098 | 1.7 | 0.66 |
| Riboflavin (mg) | -0.068 | 1.7 | 0.79 |
| Niacin (mg) | -0.246 | 25.9 | 11.77 |
| Vitamin B6 (mg) | -0.365 | 1.47 | 0.74 |
| Folic acid (μg) | -0.19 | 273 | 70.7 |
| Vitamin B12 (μg) | 0.106 | 5.15 | 2.7 |
| Vitamin C (mg) | -0.424 | 118.2 | 43.46 |
| Vitamin E (mg) | -0.419 | 8.73 | 1.49 |
| Fe (mg) | 0.032 | 13.35 | 3.71 |
| Mg (mg) | -0.484 | 310.1 | 139.4 |
| Se (μg) | -0.191 | 67 | 25.1 |
| Zn (mg) | -0.313 | 9.84 | 2.19 |
| Alcohol (g) | -0.278 | 13.98 | 3.72 |
| Caffeine (g) | -0.11 | 8.05 | 6.67 |
| Energy (kcal) | 0.18 | 2056 | 338 |

Note: RE, retinol equivalents
